# Supplementary material for: Feedback modulation of Orai1α and Orai1β protein content mediated by STIM proteins
Source: J Cell Physiol. 2024 Oct 2;240(1):e31450. doi: 10.1002/jcp.31450 (PMC11730744; doi:10.1002/jcp.31450)
Supplement: Supplementary file 1 — Supporting information. [file JCP-240-0-s001.docx]

**Feedback modulation of Orai1α and Orai1β protein content mediated by STIM isoforms**

**Joel Nieto-Felipe^†1^, Alvaro Macías-Díaz^†1^, Vanesa Jimenez-Velarde^1^, Jose J. Lopez^1^, Ginés M. Salido^1^, Tarik Smani^#2,3^, Isaac Jardin*^1^, Juan A. Rosado*^#1^**

**FIGURE S1**

**
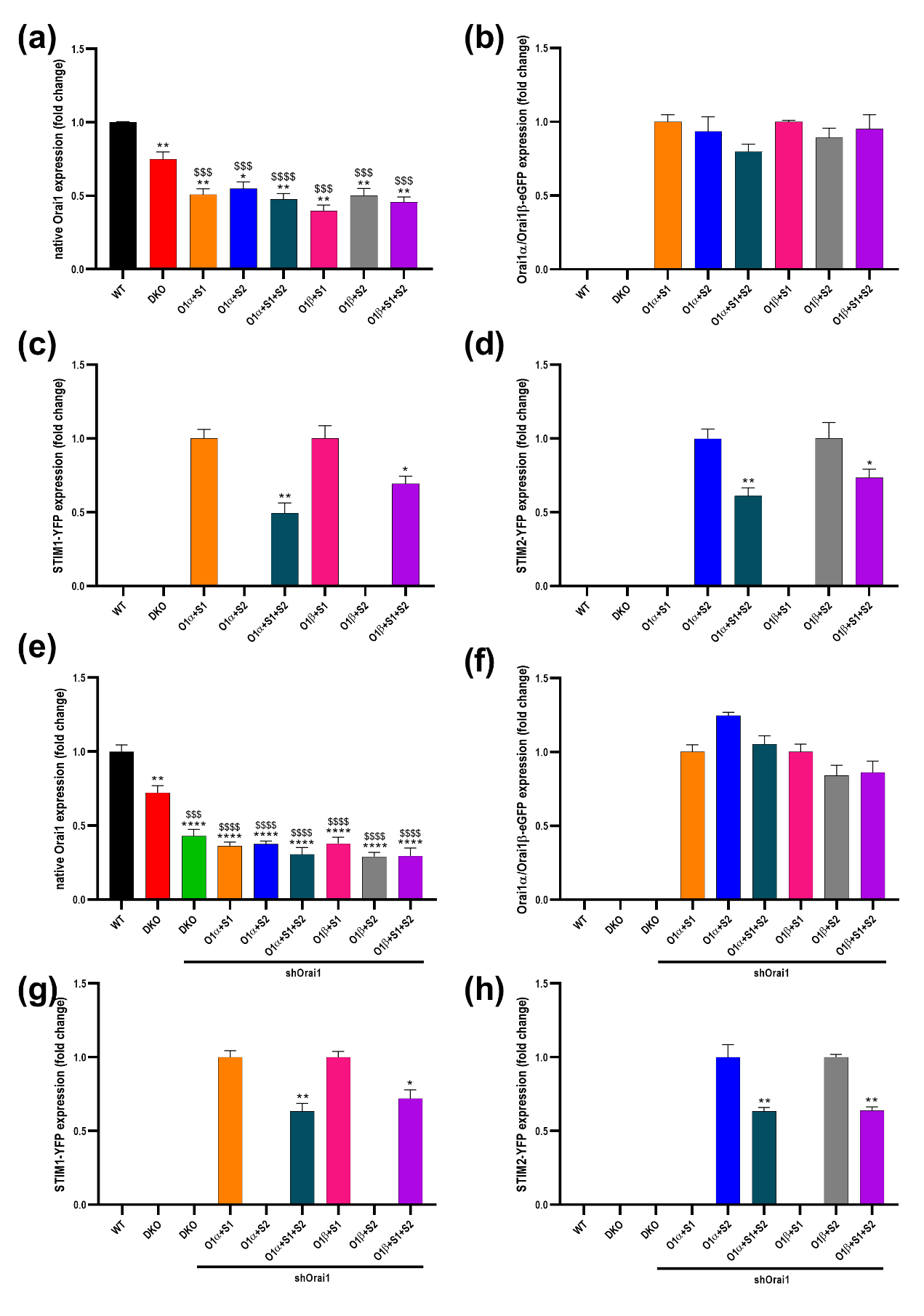
**

**FIGURE S1** Quantification of the expression of native Orai1, Orai1 variants and STIM isoforms for Figures 1f and 3f. (a-d) WT HEK-293 cells (lane 1) and STIM1,2-DKO cells either transfected with empty vector or with CMV-driven Orai1α or Orai1β in combination with either STIM1, STIM2 or both plasmids (lanes 2-8) were lysed and then subjected to 10% SDS-PAGE and Western blotting with either anti-Orai1 (C-terminal) antibody (a and b), anti-STIM1 (c) or anti-STIM2 (d) antibody, as described in material and methods. Membranes were reprobed with the anti-β-actin antibody for protein loading control. Molecular masses indicated on the right were determined using molecular-mass markers run in the same gel. n=4. Bar graphs are represented as mean ± SEM and expressed as fold change over control (first bar on the left). Data were statistically analyzed using Kruskal–Wallis test with multiple comparisons (Dunn´s test). *P < 0.05 and **P < 0.01 as compared to WT HEK-293 cells (a), Orai1α+STIM1 (c) or Orai1α+STIM2 (d). In panel a, ^$$$^P < 0.001 and ^$$$$^P < 0.0001 as compared to DKO cells. (e-h) WT HEK-293 cells (lane 1) and DKO cells (lanes 2-9) were transfected with empty vector (lanes 1 and 2), or with shOrai1 alone (lane 3) or in combination with TK-driven Orai1α or Orai1β and either STIM1, STIM2 or both plasmids (lanes 4-9), as described, and then were lysed and subjected to 10% SDS-PAGE and Western blotting with the anti-Orai1 (C-terminal) antibody (e and f), anti-STIM1 (g) or anti-STIM2 (h) antibody, as described in material and methods. Membranes were reprobed with the anti-β-actin antibody for protein loading control. Molecular masses indicated on the right were determined using molecular-mass markers run in the same gel. n=4. Bar graphs are represented as mean ± SEM and expressed as fold change over control (first bar on the left). Data were statistically analyzed using Kruskal–Wallis test with multiple comparisons (Dunn´s test). *P < 0.05, **P < 0.01 and ****P < 0.0001 as compared to WT HEK-293 cells (e), Orai1α+STIM1 (g) or Orai1α+STIM2 (h). In panel e, ^$$$^P < 0.001 and ^$$$$^P < 0.0001 as compared to DKO cells not treated with shOrai1.

**FIGURE S2**

**
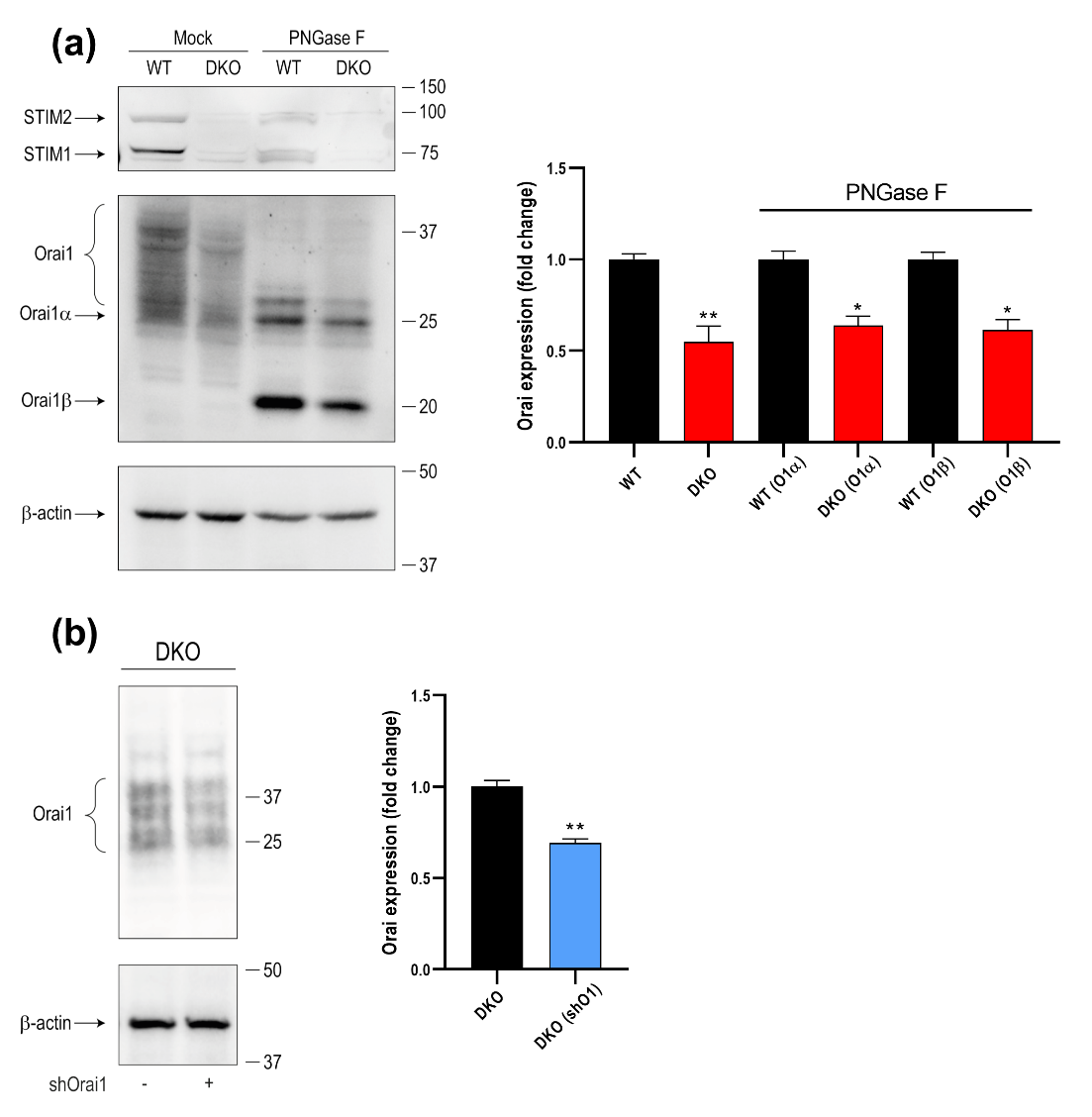
**

**FIGURE S2** Expression of STIM isoforms, Orai1 as well as Orai1α and Orai1β in WT and STIM1,2-DKO HEK-293 cells. (a) WT and STIM1,2-DKO HEK-293 cells were lysed and cell lysates were treated in the absence or presence of PNGaseF, as indicated, and then subjected to 10% SDS-PAGE and Western blot analysis using anti-STIM1 and anti-STIM2 (top panel) or anti-Orai1 C-terminal antibody (middle panel). Membranes were reprobed with anti β-actin antibody for protein loading control. Molecular masses indicated on the right were determined using molecular-mass markers run in the same gel. These results are representative of 4 separate experiments. Bar graphs represent the quantification of total Orai1, Orai1α or Orai1β in WT and DKO HEK-293 cells normalized to the β-actin expression. Data are represented as mean ± SEM and expressed as fold change relative to the expression in WT HEK-293 cells. Data were statistically analyzed using Mann-Whitney U-test. **P* < 0.05 and ***P* < 0.01 as compared to the protein expression in their corresponding WT HEK-293 cells. (b) DKO cells were either mock-transfected or transfected with shOrai1, as indicated, and then were lysed and subjected to 10% SDS-PAGE and Western blotting with anti-Orai1 (C-terminal) antibody. Membranes were reprobed with the anti-β-actin antibody for protein loading control. Molecular masses indicated on the right were determined using molecular-mass markers run in the same gel. These results are representative of 4 separate experiments. Bar graphs represent the quantification of Orai1 expression, presented as mean ± SEM and expressed as fold change (experimental/control). Data were statistically analyzed using Mann-Whitney U-test. **P < 0.01 as compared to control.

**FIGURE S3**

**
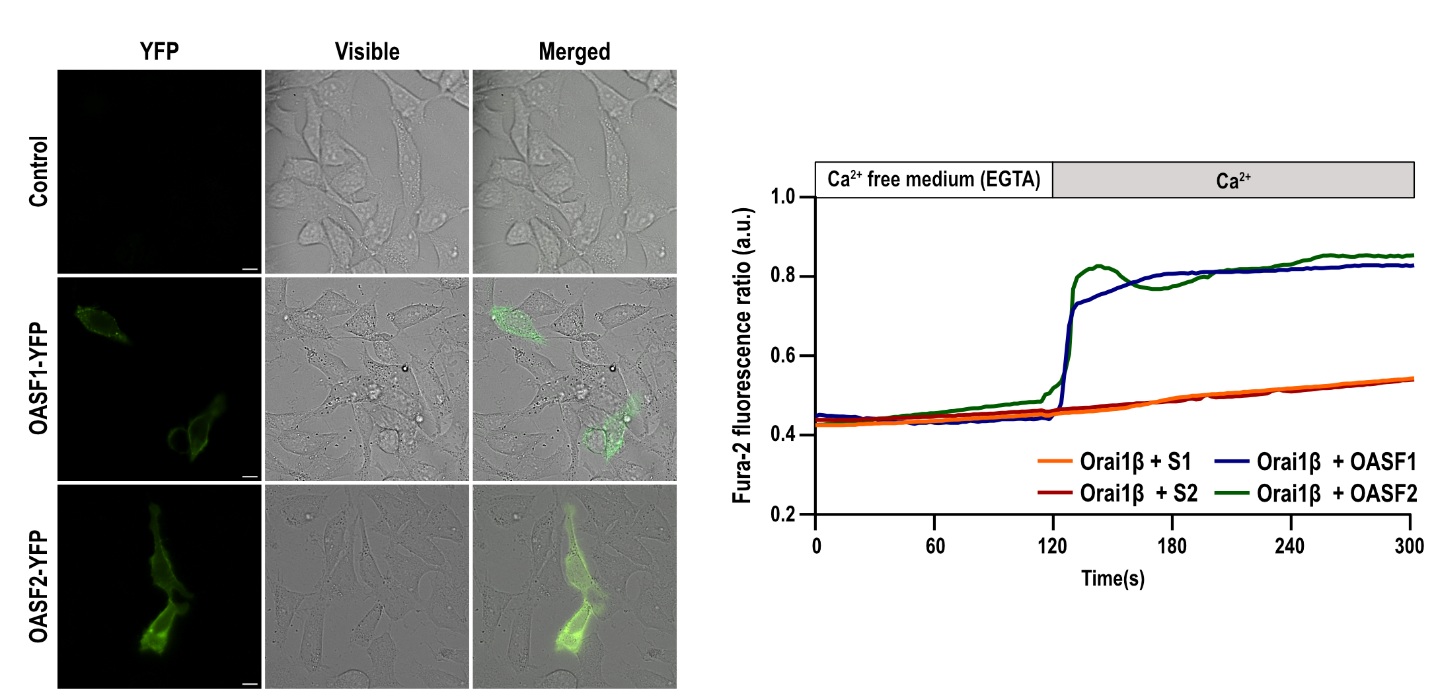
**

**FIGURE S3** Activation of constitutive Ca^2+^ influx via Orai1β by the OASF region of STIM1 and STIM2. STIM1,2-DKO HEK-293 cells were co-transfected with CMV-driven Orai1β-eGFP in combination with either STIM1-YFP, STIM2-YFP, STIM1-OASF-YFP (OASF1) or STIM2-OASF-YFP, as indicated. Forty-eight hours later GFP fluorescence was detected using an LSM900 confocal microscope. The images show representative confocal images of the STIM1-OASF of the STIM2-OASF region. The scale bar represents 15 μm. Forty-eight hours after transfection cells were loaded with fura-2 and suspended in a Ca^2+^-free HBS (100 µM EGTA added). Ca^2+^ was added to the extracellular medium at a final concentration of 1.8 mM to initiate Ca^2+^ influx.

**FIGURE S4**

**
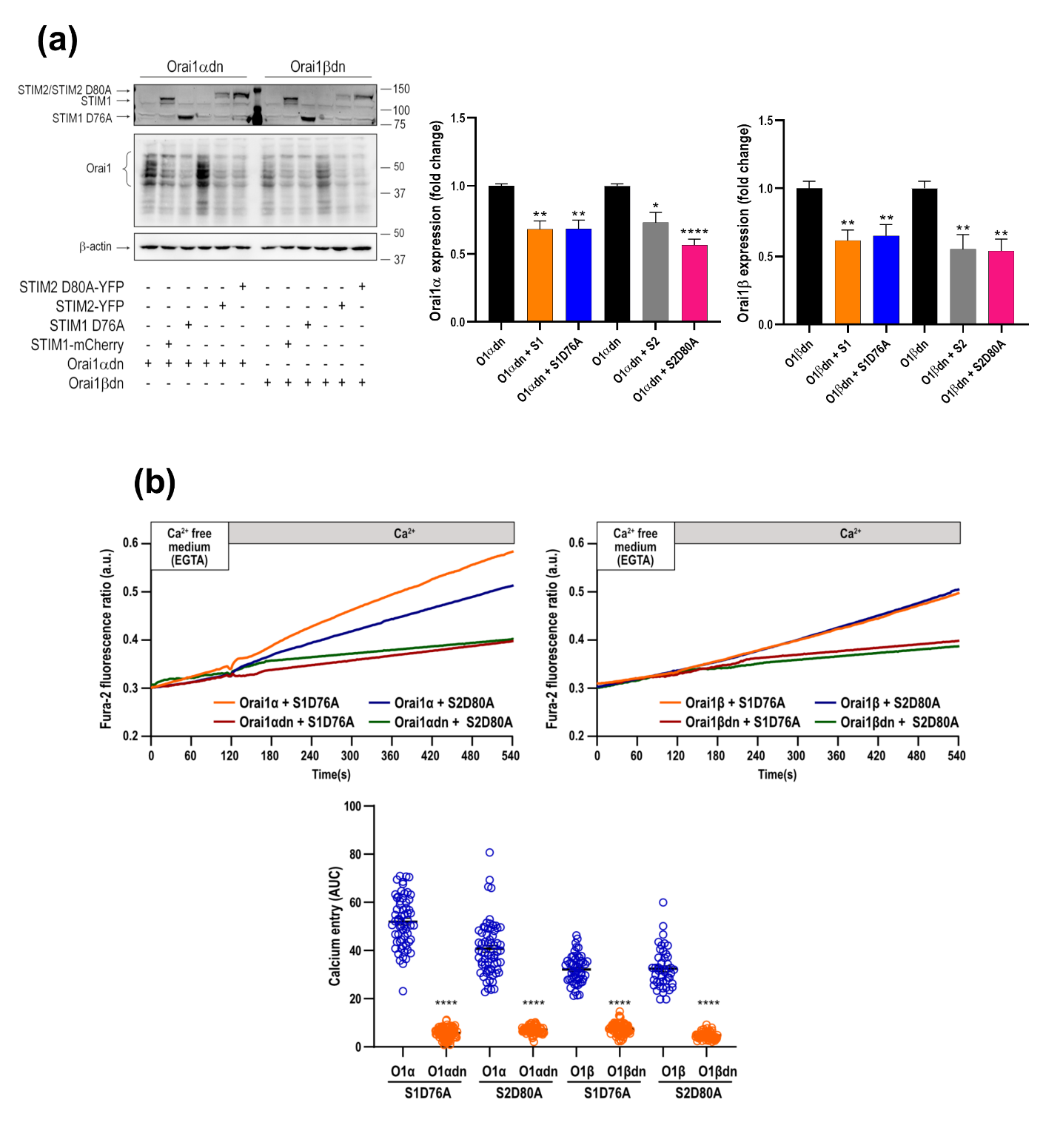
**

**FIGURE S4** Reduction of the protein content of Orai1 variants does not require Ca^2+^ influx through the channel. (a) STIM1,2-DKO HEK-293 cells were co-transfected with CMV-driven Orai1αdn or Orai1βdn alone or in combination with either STIM1-mCherry, STIM2-YFP or the STIM1-D76A and STIM2-D80A-YFP mutants, as indicated. Forty-eight hours later cells were lysed, and cell lysates were analyzed by SDS-PAGE and Western blot analysis using anti-Orai1 C-terminal antibody. Membranes were probed with anti-STIM1, anti-STIM2 or anti β-actin antibody. Molecular masses indicated on the right were determined using molecular-mass markers run in the same gel. These results are representative of 4 separate experiments. Bar graphs represent the quantification of Orai1αdn or Orai1βdn expression under the different experimental conditions normalized to the β-actin expression. Data are represented as mean ± SEM and expressed as fold change (experimental/control). Data were statistically analyzed using Kruskal–Wallis test with multiple comparisons (Dunn's test). *P < 0.05, **P < 0.01 and ****P < 0.0001 as compared to the expression of Orai1αdn or Orai1βdn in the absence of STIM. (b) STIM1,2-DKO HEK-293 cells were co-transfected with CMV-driven Orai1α-eGFP, Orai1β-eGFP or their corresponding dominant negative mutants (Orai1αdn or Orai1βdn) in combination with STIM1 D76A or STIM2-D80A mutant, as indicated. Forty-eight hours later cells were loaded with fura-2 and suspended in a Ca^2+^-free HBS (100 µM EGTA added). Ca^2+^ was added to the extracellular medium at a final concentration of 1.8 mM to initiate Ca^2+^ influx. Scatter plots represent the quantification of Ca^2+^ influx at the different experimental conditions. Data are represented as mean ± SEM. *****P* < 0.0001 as compared to their respective control.

**FIGURE S5
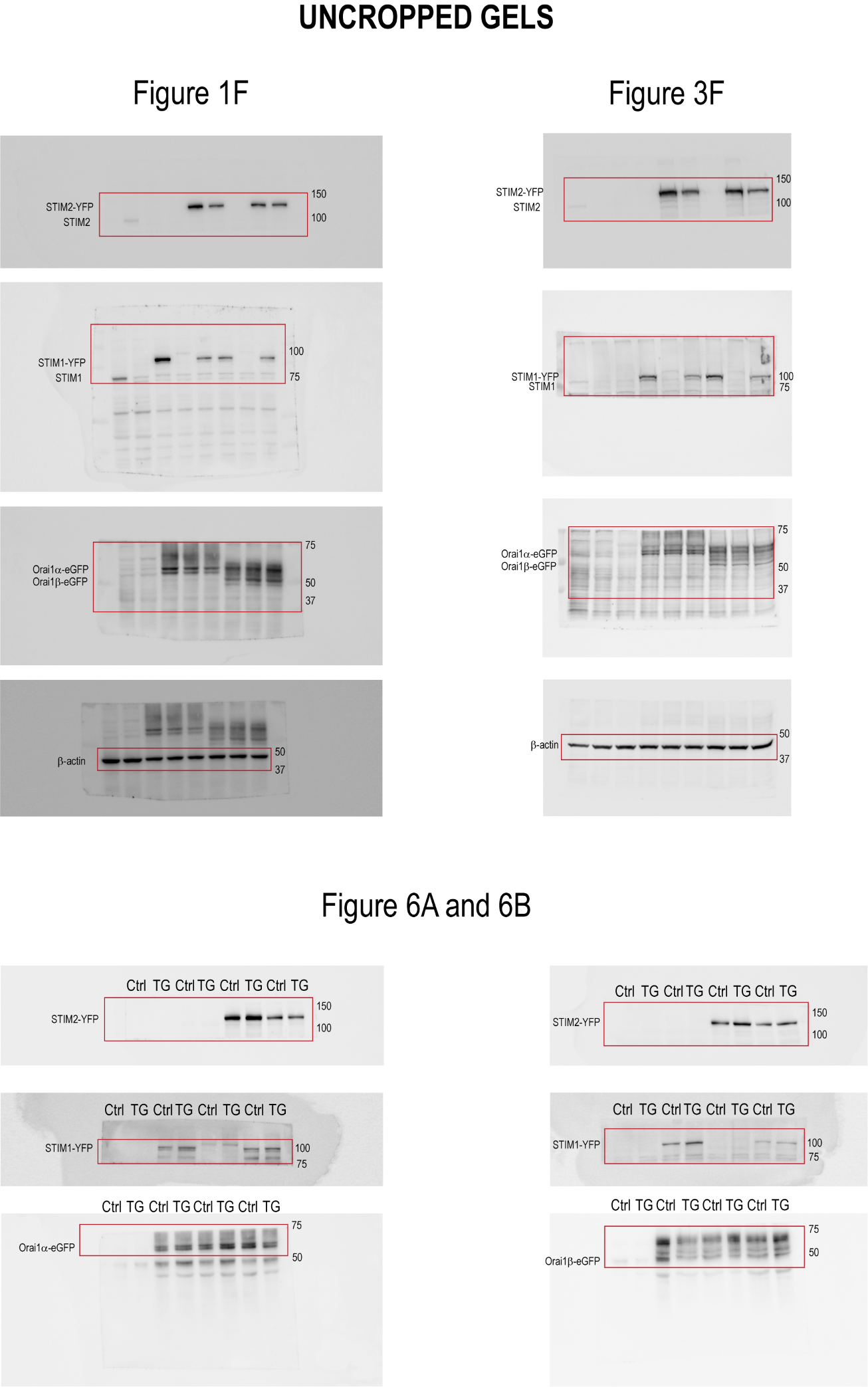
**

**Supp. Figure continue**

**
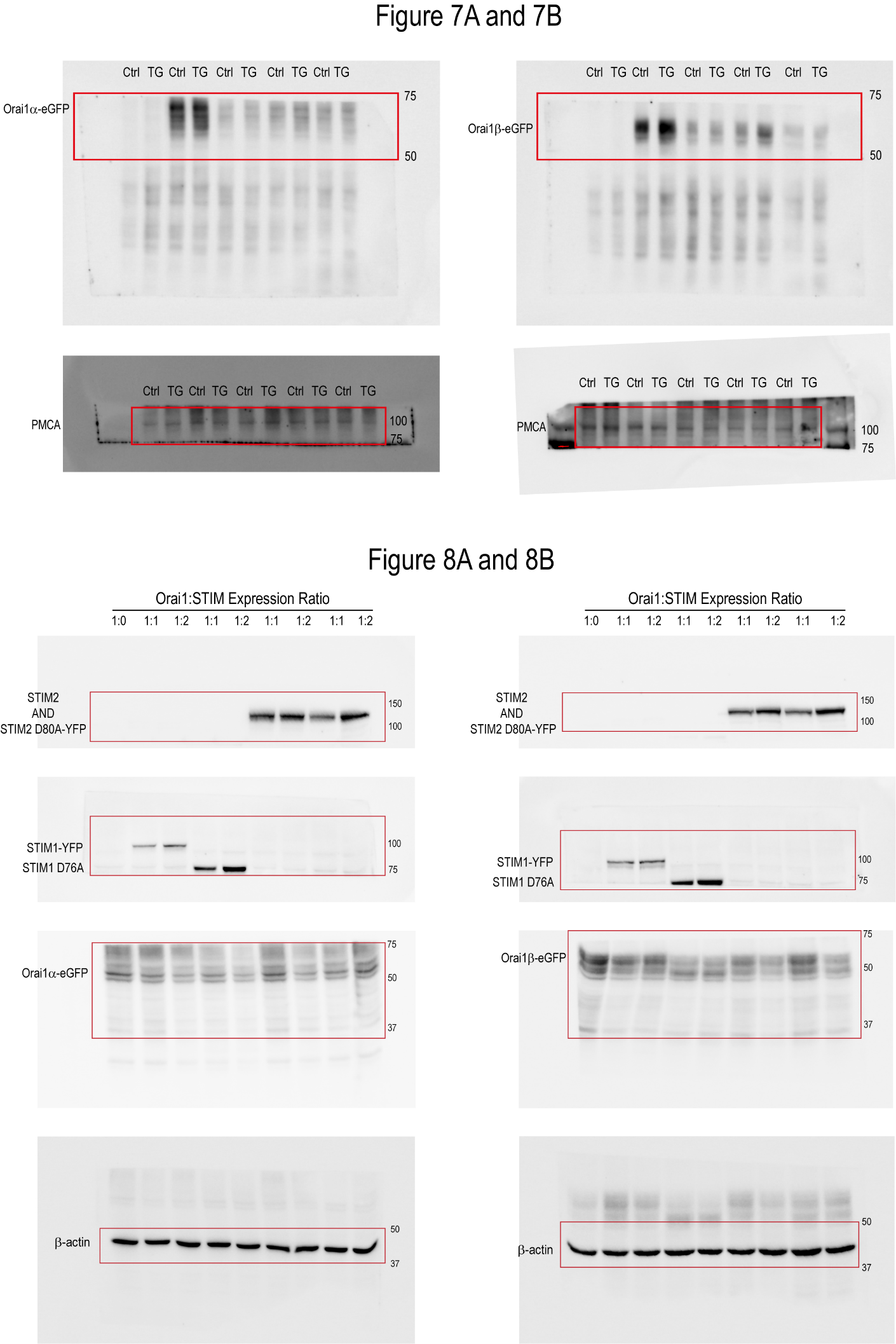
**

**Supp. Figure continue**

**
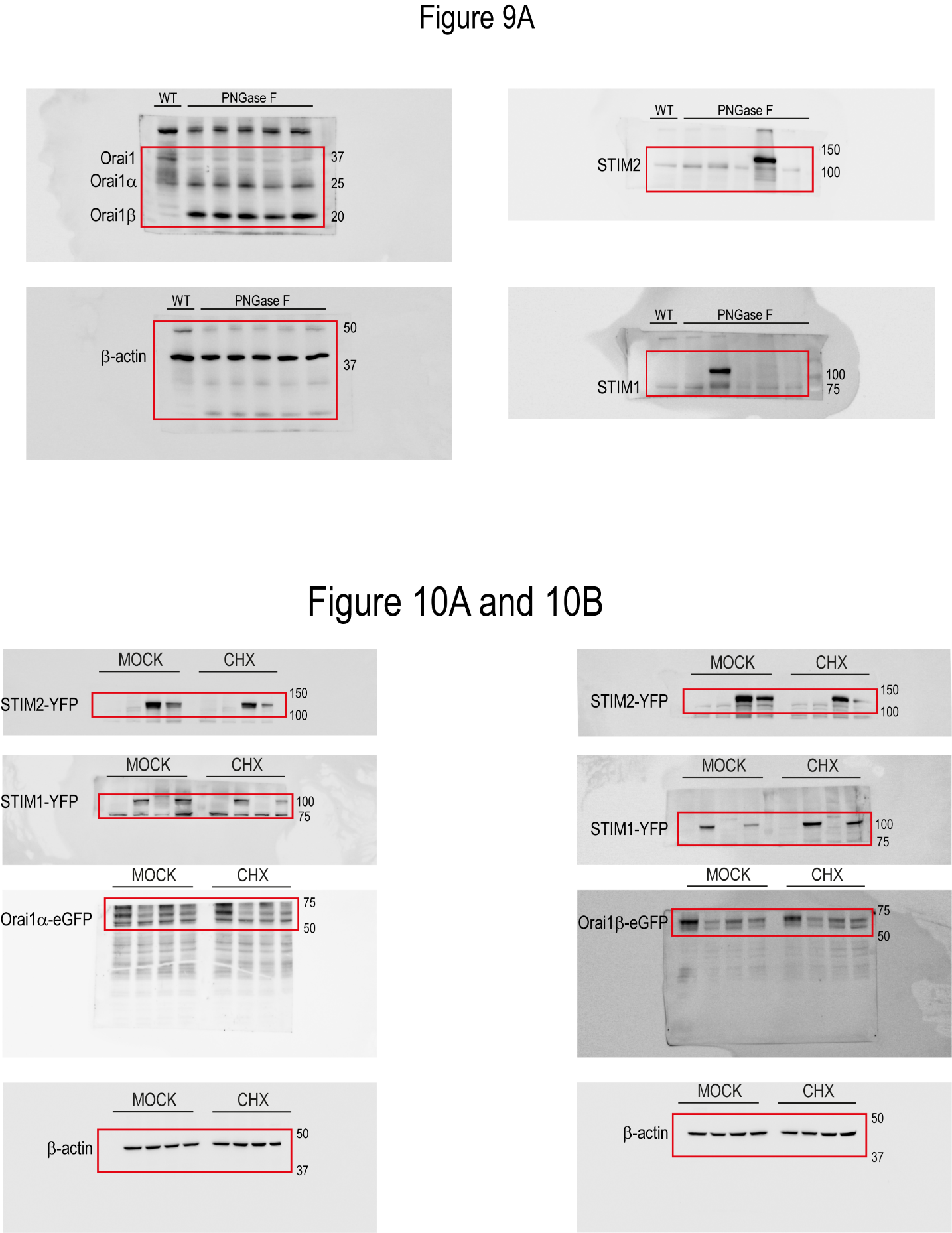
**

**Supp. Figure continue**

**
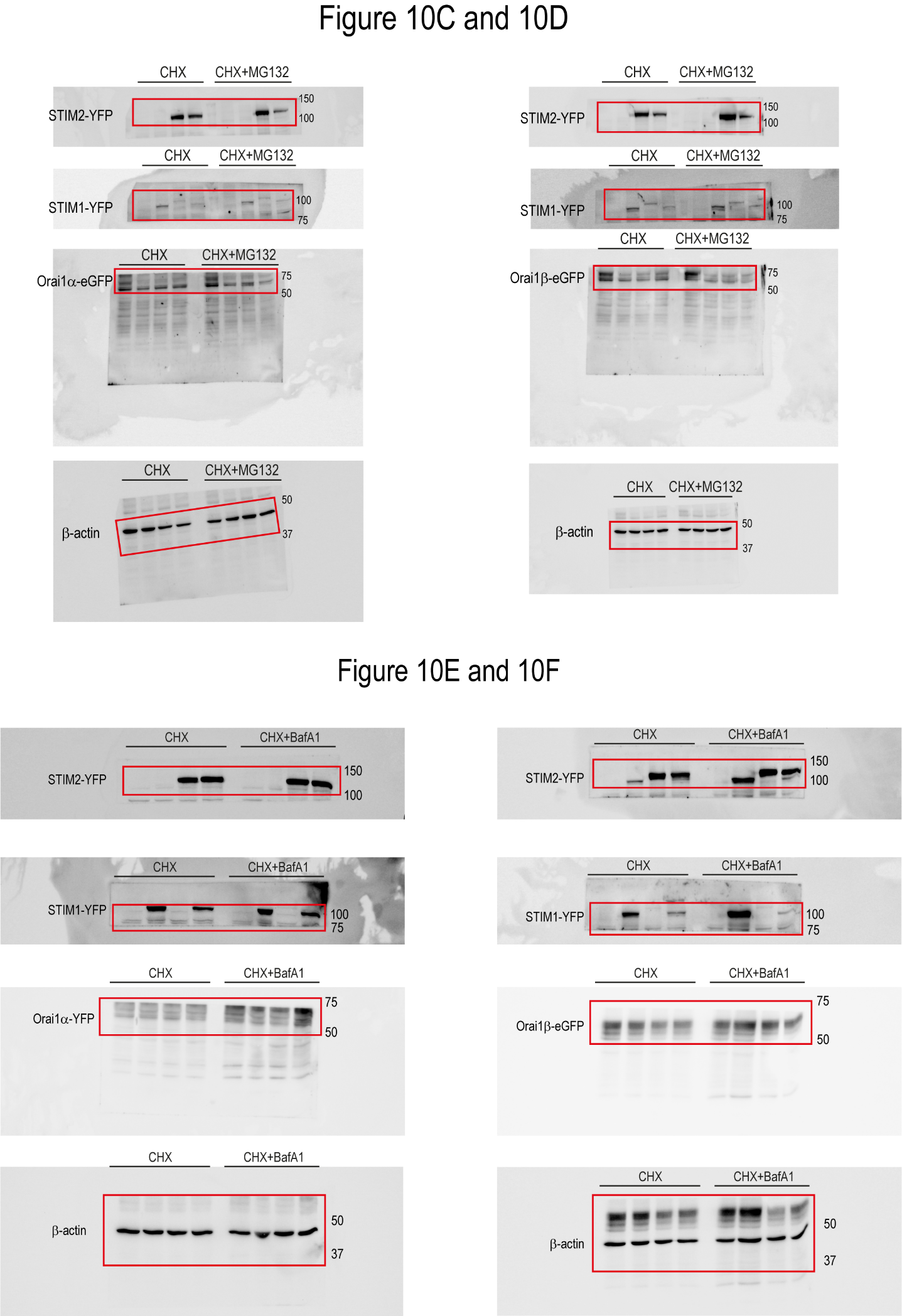
**

**Supp. Figure continue**

**
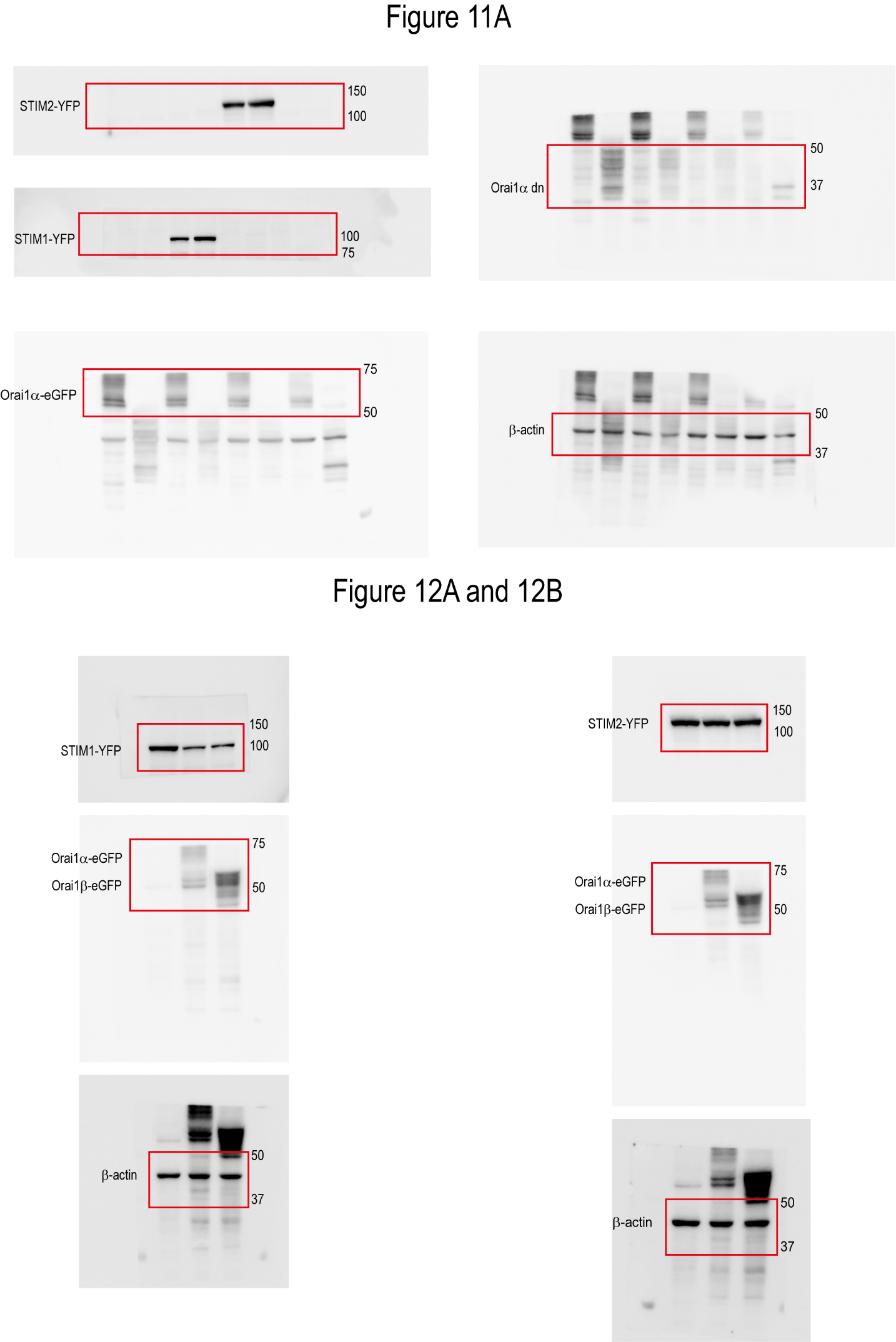
**

**Supp. Figure continue**

**
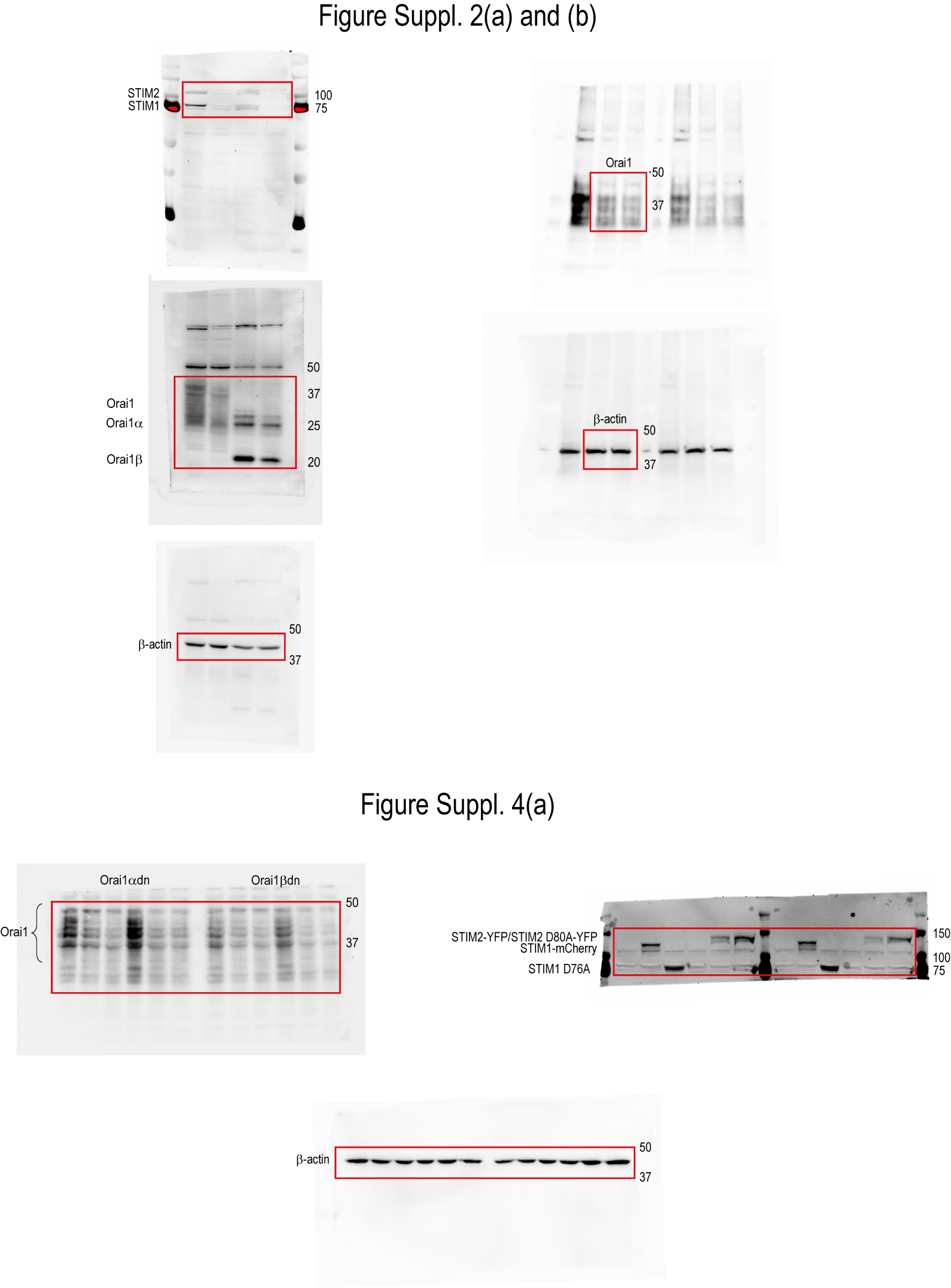
**
